# The Effect of Sulfobetaine Coating in Inhibiting the Interaction between Lyotropic Liquid Crystalline Nanogels and Proteins

**DOI:** 10.3390/gels8100653

**Published:** 2022-10-14

**Authors:** Ziqiao Zhong, Zhiwei Chen, Yuke Xie, Wenhao Wang, Zhengwei Huang, Ying Huang, Chuanbin Wu, Xin Pan

**Affiliations:** 1 College of Pharmacy, Jinan University, Guangzhou 511443, China; 2 School of Pharmaceutical Sciences, Sun Yat-sen University, Guangzhou 510006, China

**Keywords:** lyotropic liquid crystalline nanogels, interaction, sulfobetaine, adsorption mode

## Abstract

The injective lyotropic liquid crystalline nanogels (LLCNs) were widely used in drug delivery systems. But when administered in vivo, LLCNs exposed to the biological environment interact with proteins. Recently, it has been shown that nanoparticles coated with zwitterions can inhibit their interaction with proteins. Thus, in this study, the interaction between proteins and LLCNs coated with the zwitterionic material sulfobetaine (GLLCNs@HDSB) was investigated using bovine serum albumin (BSA) as a model protein. Interestingly, it was found that GLLCNs@HDSB at higher concentrations (≥0.8 mg/mL) could block its interaction with BSA, but not at lower concentrations (<0.8 mg/mL), according to the results of ultraviolet, fluorescence, and circular dichroism spectra. In the ultraviolet spectra, the absorbance of GLLCNs@HDSB (0.8 mg/mL) was 1.9 times higher than that without the sulfobetaine coating (GLLCNs) after incubation with protein; the fluorescence quenching intensity of GLLCNs@HDSB was conversely larger than that of the GLLCNs; in circular dichroism spectra, the ellipticity value of GLLCNs@HDSB was significantly smaller than that of the GLLCNs, and the change in GLLCNs@HDSB was 10 times higher than that of the GLLCNs. Generally, nanoparticles coated with sulfobetaine can inhibit their interaction with proteins, but in this study, LLCNs showed a concentration-dependent inhibitory effect. It could be inferred that in contrast to the surface of nanoparticles covered with sulfobetaine in other cases, the sulfobetaine in this study interacted with the LLCNs and was partially inserted into the hydrophobic region of the LLCNs. In conclusion, this study suggests that coating-modified nanoparticles do not necessarily avoid interacting with proteins, and we should also study coating-modified nanoparticles interacting with proteins both in vitro and in vivo. In the future, finding a coating material to completely inhibit the interaction between LLCNs and proteins will generate a great impetus to promote the clinical transformation of LLCNs.

## 1. Introduction

In recent years, nanodrug delivery systems (NDDS) have been widely reported and applied due to their unique advantages, such as targeting effects, sustained and controlled release effects, and fewer toxic side effects [1]. Indeed, several NDDS, such as PEGylated liposomal doxorubicin injection (LipoDox^®®^) and nanosized albumin-bound paclitaxel injection (Abraxane^®®^) [2], have been applied in clinical practice. However, except for a few that have been used clinically, most NDDS are still under preclinical development because they do not respond well to treatment when administered in vivo. The effectiveness of NDDS may be impacted by several factors, including the characteristics of nanoparticles themselves [3] and their interaction with biological systems [4,5]. In biological fluids, the administered NDDS will interact with a variety of biological macromolecules (including proteins, DNA, lipids, and carbohydrates). The interaction of NDDS with proteins in the biological environment can be controlled by both electrostatic and non-electrostatic interactions [6]. Along with such an interaction, the release behavior [7], pharmacokinetic profile [8], and toxicity of NDDS [9] will all be significantly impacted. For example, Huang et al., measured the electrophoretic mobility and aggregation rate constant of silica particles coated with lysozymes, and the adsorbed amount of lysozymes on the silica. They found that lysozymes enhance the aggregation of silica when the lysozyme-coated silica is near the isoelectric point [10].

**Figure 1:**
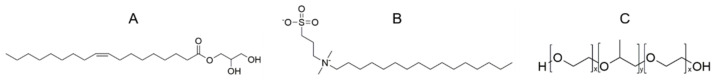
The structure of chemicals: (**A**) glycerol monooleate; (**B**) 3-(Hexadecyldimethylammonio) propane-1-sulfonate; (**C**) poloxamer formula: x and y are the lengths of polyepoxyethane (PEO) and polyepoxypropane (PPO) chains; the weight ratio of oxirane to 1,2-epoxypropane in poloxamer 407 is 7:3.

According to the above, it is not difficult to see that the clinical transformation of NDDS will be boosted by inhibiting the interaction between NDDS and biomolecules. A reasonable strategy is to construct a physical barrier between both counterparts by a coating layer. Modification with polyethylene glycol (PEG), viz. PEGylation, is frequently employed to decrease nonspecific protein adsorption and increase nanoparticle half-lives; however, the efficacy is not always satisfactory [11,12,13]. Zwitterionic components with neutral overall charges have recently been suggested as PEG alternatives. Manon et al., demonstrated that coating nanoparticles with zwitterionic polymers enable partial to virtually complete elimination of protein adsorption [14]. The interaction between zwitterionic-coated NDDS and biomolecules is poorly characterized, compared to PEGylated ones, which prevents further biomedical uses. To determine if zwitterionic-coated NDDS can diminish their adsorption to proteins, sulfobetaine (HDSB, 3-(Hexadecyldimethylammonio) propane-1-sulfonate, Figure 1B) was chosen as the surface coating in this study.

Lyotropic liquid crystalline nanogels (LLCNs) were selected as the model NDDS for interacting with biological macromolecules, for there were few studies on the interaction between LLCNs and protein so far. LLCNs refer to the spontaneous self-assembly of lipids with amphiphilic materials in an aqueous solution into a closed lipid bilayer “honeycomb” structure containing bicontinuous water and lipid channels [15]. This system takes the cubic lattice as the structural unit, extends in three dimensions in space, and the lipid bilayer is twisted into a compact structure with a cyclic arrangement and minimum surface area [16,17]. LLCNs are usually composed of lipids, stabilizers, and water. Additionally, glycerol monooleate (GMO, Figure 1A) and the stabilizer poloxamer 407 (F127, Figure 1C) are most frequently utilized. In this study, GMO was chosen as the lipid component, and F127 as the stabilizer.

As an injection drug delivery system, LLCNs have many advantages, such as high surface area, sufficient opportunity for functionalization, and a bioadhesive nature [18]. LLCNs are an intermediate phase structure used in the fields of biological imaging, therapeutic diagnosis, and drug targeting. The adjustable internal structure and external surface pave the way for better delivery of both hydrophobic and hydrophilic molecules [19,20]. However, as LLCNs often interact with proteins in vivo, their clinical transformation is delayed. Noticeably, the new strategy of coating LLCNs with HDSB may inhibit the interaction with protein and promote the clinical application of LLCNs. To investigate the interaction between LLCNs and proteins with or without the HDSB coating, the model protein must be selected.

Bovine serum albumin (BSA) was selected as the model protein in many studies because the sequence similarity and homology between human serum albumin (HSA) and BSA are quite high (approximately 83% and 72%, respectively) [21,22]. For example, Fu et al., chose Solutol^®®^ HS 15 and BSA as the model nanomicelles and model protein, respectively, to investigate the interaction and the time evolution between protein and nanomicelles and to further understand the interaction mechanisms [23]. Aleksandra M. et al., investigated the interaction between L-methionine-capped silver nanoparticles (AgMet), and bovine serum albumin (BSA) to predict the fate of AgMet after its contact with the most abundant blood transport protein [24]. Wang et al., chose BSA, Lysozyme, and Bovine hemoglobin as model proteins to investigate the protein corona formation process of Soluplus^®®^ nanomicelles, and they found two modes of interaction between nanoparticles and proteins [25]. Thus, BSA was chosen as the model protein in this study. The basic physicochemical information about BSA is shown in Table 1 [25].

In the presented work, the hydrodynamic diameter (*D_H_*) and Zeta potential of LLCNs with or without HDSB coating were characterized. The interaction mechanism between BSA and LLCNs with or without HDSB coating was revealed and discussed from the point of view of protein properties by measuring ultraviolet-visible (UV-Vis), fluorescence, and circular dichroism (CD) spectra. We believe that this study will provide new insights for promoting the clinical transformation of LLCNs.

## 2. Results and Discussion

### 2.1. Particle Size and ζ-Potential of GLLCNs and GLLCNs@HDSB

To confirm that HDSB was successfully coated on the surface of the GLLCNs, the particle sizes of the nanogels with and without HDSB were determined. It was expected that when HDSB was successfully coated, the surface properties of GLLCNs would change to that of HDSB.

As shown in Table 2, the particle size of the GLLCNs was 135.30 ± 3.84 nm, and the polydispersity index (PDI) was less than 0.25, which indicated that the size distribution of nanogels was narrow [25]. The particle size of GLLCNs@HDSB was 207.10 ± 2.14 nm, and PDI was also less than 0.25. It was obvious that the GLLCNs’ particle size was raised after HDSB was applied, proving that GLLCNs@HDSB had been properly prepared. Similarly, the potentials of GLLCNs and GLLCNs@HDSB were measured, as shown in Table 2. The GLLCNs had a potential of −16.00 ± 0.26 mV, while the GLLCNs@HDSB had a potential of −1.65 ± 0.05 mV. It was obvious that the potential of the GLLCNs@HDSB was much higher than that of the GLLCNs. This was because HDSB was electrically neutral and GLLCNs coated with HDSB had surface characteristics that are comparable to those of HDSB [26]. The shift in potential further supported the assertion that HDSB was successfully coated on the surface of GLLCNs@HDSB.

### 2.2. UV-Vis Spectra of GLLCNs and GLLCNs@HDSB after Incubation with BSA

UV-Vis spectra were used to determine the microenvironment change of BSA interacting with GLLCNs and GLLCNs@HDSB. The UV-Vis absorption of BSA is mainly derived from tyrosine (Tyr) and tryptophan (Trp) residues. For hydrophobic amino acid residues such as Tyr and Trp, the change in their distribution microenvironment polarity can be reflected by the change in their inherent UV-Vis spectral properties [27]. Therefore, the absorption spectra of BSA incubated with GLLCNs and GLLCNs@HDSB at 200–800 nm were measured, and the absorbance value (Abs) and the peak absorption wavelength were analyzed to explore the interaction between GLLCNs and GLLCNs@HDSB with BSA.

**Figure 2:**
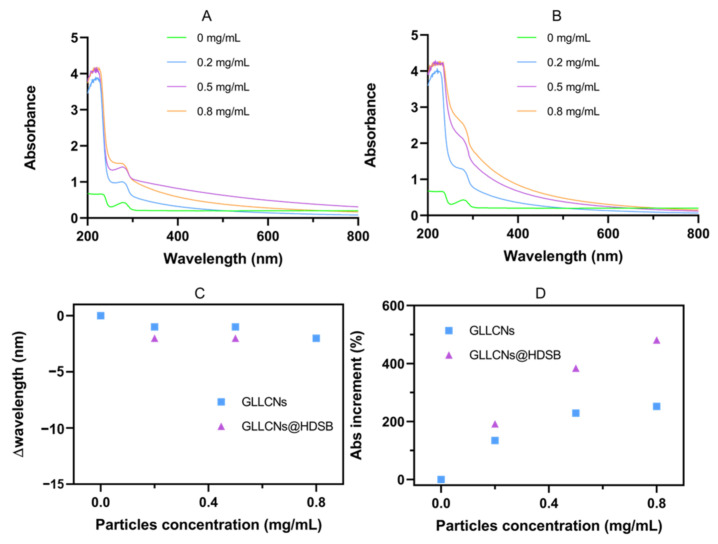
(**A**,**B**) UV-Vis spectra of BSA incubated with GLLCNs and GLLCNs@HDSB at different concentrations. (**C**) The Δwavelength of GLLCNs and GLLCNs@HDSB. (**D**) The Abs increment of GLLCNs and GLLCNs@HDSB.

As shown in Figure 2A–D, the Abs of BSA rose with increasing concentrations of GLLCNs and GLLCNs@HDSB, but the rise was larger in the GLLCNs@HDSB group, especially at higher concentrations. The hyperchromic shift for proteins might be correlated to the partial exposure of Trp residues induced by the protein-nanogels interactions. At a low concentration, the increase of Abs in the GLLCNs group and GLLCNs@HDSBs group was the same, but with the increase in concentration, the increase of Abs in the GLLCNs@HDSB group was more pronounced. When the concentration was 0.8 mg/mL, the Abs in the GLLCNs@HDSB group was 1.9 times that in the GLLCNs group. Trp was sharply exposed on the surface of the nanogels, which might be induced by the interaction between negatively charged BSA with HDSB-quaternary ammonium ions in HDSB through electrostatic force.

### 2.3. Fluorescence Spectra of GLLCNs and GLLCNs@HDSB after Incubation with BSA

**Figure 3:**
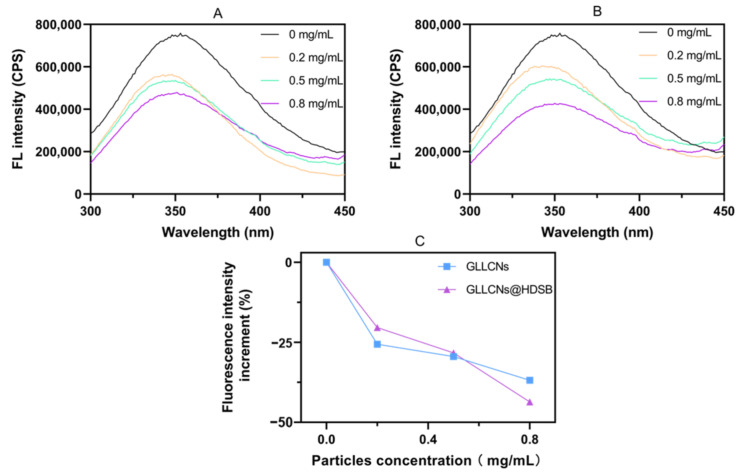
(**A**,**B**) Fluorescence spectra of BSA incubated with GLLCNs (**A**) and GLLCNs@HDSB (**B**) at different concentrations. (**C**) The peak fluorescence increment of BSA incubated with GLLCNs and GLLCNs@HDSB at different concentrations.

Moreover, to confirm the interaction between the GLLCNs and GLLCNs@HDSB with BSA, the fluorescence spectra of BSA incubated with 0.2, 0.5, and 0.8 mg/mL GLLCNs and GLLCNs@HDSB for 2 h were determined. As shown in Figure 3A,B, fluorescence quenching occurred to varying degrees in both GLLCNs and GLLCNs@HDSB groups, and the quenching effect was enhanced with the nanogel concentration increase. It is believed that the interaction between proteins and nanogels is proportional to the fluorescence quenching degree. It was interesting to note that, as shown in Figure 3C, the fluorescence quenching degree of the GLLCNs@HDSB group was weaker than that of the GLLCNs group when the concentration was low (≤0.5 mg/mL), but when the concentration of nanogels was 0.8 mg/mL, the fluorescence quenching intensity of GLLCNs@HDSB group was conversely larger than that of GLLCNs group. This suggested that, at high concentrations, the HDSB-coated GLLCNs could inhibit the adsorption of BSA, whereas at low concentrations, there was either no such inhibitory effect or the inhibitory effect was not readily apparent.

However, the fluorescence correlation spectroscopy (FCS) made by Ashraf et al., pointed to negligible adsorption of the model human serum albumin onto quantum dots (QDs) coated with monomeric sulfobetaine-based ligands [28]. The results of the fluorescence spectra in this study were inconsistent with the findings of Ashraf et al. The reason will be discussed later.

### 2.4. CD of GLLCNs and GLLCNs@HDSB following BSA Incubation

**Figure 4:**
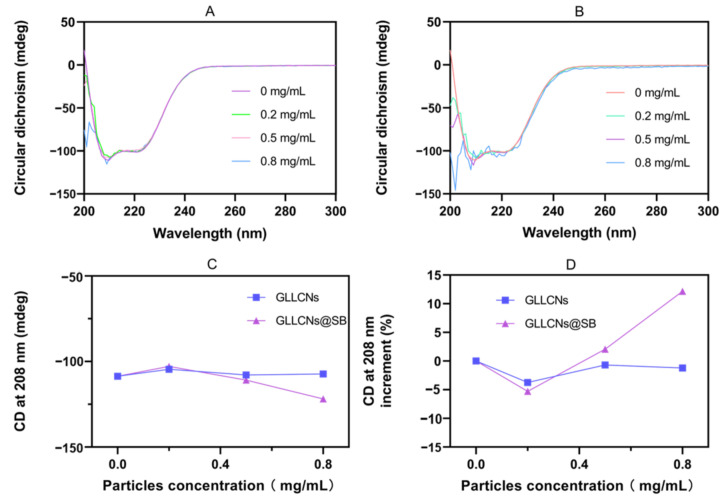
(**A**,**B**) CD spectra of BSA incubated with GLLCNs (**A**) and GLLCNs@HDSB (**B**) at different concentrations. (**C**) The CD ellipticity values at 208 nm of different particles. (**D**) The ellipticity values in increasing increments of GLLCNs and GLLCNs@HDSB.

CD spectra were used to explore the conformational changes of BSA when interacting with LLCNs. According to the literature, the CD spectrum of free BSA solution has two negative peaks at 209 and 220 nm, which represent the characteristic α-helix and n-π* electron transition of the BSA peptide sequence [29]. In this study, the CD spectrum of BSA showed double negative peaks at 208 and 222 nm (Figure 4A,B). The ellipticity value at 208 nm was summarized in Figure 4C. It could be seen that there was no significant difference in conformational change between GLLCNs and GLLCNs@HDSB at a lower concentration (≤0.5 mg/mL), but the ellipticity value of the GLLCNs@HDSB was significantly smaller than that of the GLLCNs when the concentration of nanogels was 0.8 mg/mL, and the change in the GLLCNs@HDSB group was 10 times higher than that of the GLLCNs group, which indicated that the interaction between GLLCNs@HDSB and BSA was stronger, and the conformational change of BSA was more obvious when the concentration was higher. This was consistent with the results of the UV-Vis spectrum and fluorescence spectrum. It is worth noting that the delicate determination of the secondary structure of BSA, including α-helix, β-folding, β-rotation, Ω-ring, and random crimp, is still in progress and will be reported in the future.

### 2.5. Analysis of the Interaction Modes

As for the above research results, a summary has been made, as shown in Table 3. It was shown that the interaction between GLLCNs@HDSB and BSA was concentration-dependent.

The conventional zwitterion-coated nanoparticles reduce their interactions with proteins. For example, Estephan et al., prepared zwitterionic-coated nanoparticles and mixed them with salt, serum, lysozyme, and serum albumin and found that this type of surface modification was highly effective in preventing protein adsorption [30]. Similarly, Manon et al., utilized fluorescent core/multishell CdSe/CdS/ZnS quantum dots coated with zwitterionic polymer ligands to investigate the ability of zwitterion-coated nanoparticles to inhibit protein adsorption in complex biological media. The results showed that there was no protein corona around the HDSB-coated nanoparticles at all [14]. However, interestingly, unlike the traditional zwitterion-coated nanoparticles, which could reduce protein adsorption, GLLCNs@HDSB did not inhibit its interaction with protein at lower concentrations but showed a certain ability to inhibit protein interaction at higher concentrations.

**Figure 5:**
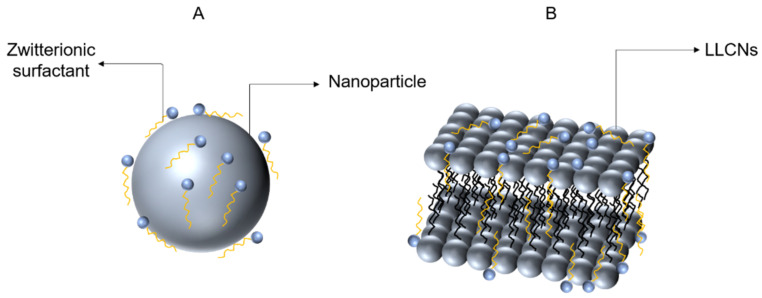
(**A**) The surface coating mode; (**B**) the insertion mode.

For the phenomena shown in this study, we offer the following explanations. Firstly, in other studies, the coating material had little interaction with the NDDS per se and was, therefore, likely to be perfectly coated on the surface, such as the nanoparticles prepared by Estephan et al. and Manon et al., mentioned above. We would like to name the interaction mode between such coating substances and nanogels the surface coating mode, as shown in Figure 5A. However, in this study, HDSB may interact with the GLLCNs, and the long adipose chain of HDSB may be inserted into the hydrophobic region of the GLLCNs without that perfect coating on the surface of the GLLCNs (called the insertion mode, as shown in Figure 5B); thus, the effect of GLLCNs@HDSB’s resistance to protein becomes poor. In most cases, the coating materials perfectly covered the surface of nanoparticles. For example, in Alallam et al.’s study, PEG coating was mainly distributed on the surface of the nanoparticles [31]. However, different coating modes were also observed. Alallam et al., comparatively studied three different polymer coatings, viz., chitosan (CS), gum Arabic (GA), and PEG, to improve the efficacy of plasmid-loaded alginate nanoparticles. They found that the three coating polymers increased the size differently, which could be due to the different coating modes around the surface of the nanoparticles. PEG coating molecules mostly adhered to the nanoparticles’ surface and did not completely diffuse into the alginate network, while CS could electrostatically attract negatively-charged alginate due to its short chain [32]. Moreover, the GA polymer chain could attract negatively-charged alginate molecules with electrostatic forces as its ampholytic polymer. With regards to this study, the HDSB coating did not simply coat the surface of the LLCNs but instead provoked an interaction. This was similar to the study of Alallam et al., in which CS and GA coatings produced an interaction with nanoparticles. Secondly, as a zwitterionic surfactant, HDSB has a strong interaction with BSA that is also surface-active under certain conditions. At low concentrations, the interaction was not strong enough, but at high concentrations, the interaction became stronger. Specifically, the interaction between GLLCNs@HDSB and BSA was not obvious at low concentrations, but strong at high concentrations. Likewise, Leonardo et al., found that the interaction between the zwitterionic surfactant N-hexadecyl-N, N-dimethyl-3-ammonio-1-propanesulfonate (HPS) and the giant extracellular hemoglobin of Glossoscolex Paulus (HbGp) depended largely on concentration [33].

### 2.6. Inspiration for Future Application

LLCNs have been widely reported for drug delivery and have a broad application prospect. For instance, He et al.’s studies of curcumin-loaded lipid cubic liquid crystalline nanogels revealed that these nanogels had high entrapment efficiency and continuous release, which significantly boosts the bioavailability of oral curcumin [34]. Additionally, the LLCNs containing vitamin B12 prepared by Maiorova et al., could improve the targeted delivery and induced release of drugs [35]. Similarly, Mahmoud et al., created an ocular gel containing ketoconazole cubic liquid crystal nanogels, which had enhanced permeability, ocular availability, and antifungal activity.

Theoretically, a zwitterionic coating could inhibit the interaction of nanogels with proteins in the surrounding environment. Therefore, we considered using a more used zwitterion-coating material HDSB to carry out this study. However, our current study showed that under the existing experimental conditions, the HDSB coating did not inhibit the interaction between LLCNs and BSA at low concentrations but showed a certain degree of inhibition at high concentrations. This suggested that a certain coating material is not suitable for all types of nanoparticles, and the coating strategy needs to be further explored. We should develop coating strategies specifically for LLCNs or other nanomaterials to promote the clinical application of LLCNs or other nanomaterials. In the future, we will screen coating methods that can effectively inhibit the interaction between LLCNs and proteins to promote the clinical application of LLCNs.

## 3. Conclusions

In this study, two kinds of nanogels were prepared: GLLCNs and GLLCNs@HDSB, where the surface of GLLCNs@HDSB was coated with HDSB. Then, the two kinds of nanogels were characterized by particle size and zeta potential. To explore the interaction of GLLCNs and GLLCNs@HDSB with BSA, a series of spectral tests were carried out, and the UV-Vis, fluorescence, and CD spectra showing the interaction between GLLCNs and GLLCNs@HDSB with BSA were obtained. In the UV-Vis spectra, it could be seen that the BSA Abs incubated with GLLCNs and GLLCNs@HDSB rose with the increase in concentration, but the increase in GLLCNs@HDSB was larger than that of GLLCNs at the higher concentration, indicating that the interaction between GLLCNs@HDSB and BSA was stronger when the concentration was higher. In addition, it was also observed in the fluorescence spectrum that when the concentration of nanogels was 0.8 mg/mL, the fluorescence quenching degree of GLLCNs@HDSB was greater than that of GLLCNs, and the interaction between the nanogels and BSA was stronger, indicating that HDSB coating could inhibit protein adsorption when the concentration increased to a certain extent. The results of the CD also confirmed this point.

However, the results of this study are different from other studies on HDSB-coated nanogels. We hypothesized that this might be because HDSB interacts with LLCNs, and HDSB does not simply wrap around the surface of LLCNs, but rather the long fatty chains of HDSB are inserted into the hydrophobic region of the LLCNs, which affects the ability of HDSB to inhibit protein interactions. In addition, it may also be related to the concentration of GLLCNs@HDSB.

In conclusion, LLCNs with HDSB coating could not sufficiently inhibit their interaction with proteins. This study proposes a warning for nanomedicine investigators: The coating does not necessarily inhibit the interaction between nanoparticles and proteins. This suggests that the fate of nanoparticles in vivo and in vitro should be investigated, with or without coating, rather than merely forecasting the in vivo fate by the in vitro performance. For LLCNs, even in the presence of coatings, we should consider that the protein inhibition effects can be dissatisfactory and, hence, investigate their interactions with proteins in depth. Moreover, if a suitable coating material can be found to significantly or completely inhibit the interaction between LLCNs and proteins, it will greatly promote the clinical transformation process of LLCNs.

## 4. Materials and Methods

### 4.1. Materials

GMO was provided by Danisco (Copenhagen, Denmark). F127 was purchased from Aikeda Chemical Reagent Co., Ltd. (Chengdu, China). HDSB was purchased from China National Pharmaceutical Group Corporation (Beijing, China). BSA was purchased from neoFroxx GmbH (Einhausen, Germany). Ultra-pure water was obtained by the VEOLIA-ELGA system (Veolia, UK).

### 4.2. Preparation and Characterization of LLCNs

GMO (90 mg) was added to a 50 mL centrifuge tube containing 1 mL ethanol, and 10 mL of 1 mg/mL aqueous Poloxamer 407 solution was slowly added during the stirring process. Then, the tube was stirred in a 75 °C water bath for 50 min. Lastly, the obtained system was ultrasonically crushed at a frequency of 6 s onset with 3 s interval for a total of 6 min, and GMO lyotropic liquid crystalline nanogels (GLLCNs) were harvested. The GLLCNs coated with HDSB (GLLCNs@HDSB) were produced by mixing the GLLCNs solution with 11 mL of an aqueous solution containing 75 mg of HDSB for 1 h in an ice bath. The generated GLLCNs and GLLCNs@HDSB were 80 times diluted. Then, the prepared nanogels were subjected to size, PDI, and zeta potential determination using a Malvern Mastersizer 2000^®®^ and Zetasizer (Malvern Instruments Ltd., Malvern, UK). The data were measured in the cuvette equilibrated at 25 °C prior to analysis.

### 4.3. The Interaction between GLLCNs and GLLCNs@HDSB with BSA

Several spectroscopic techniques were applied to examine the interaction between GLLCNs and GLLCNs@HDSB with BSA. The GLLCNs solution and the GLLCNs@HDSB solution were added to BSA at doses of 0.2, 0.5, and 0.8 mg/mL, respectively, and incubated for 2 h. The UV-Vis spectra were recorded from 200 to 800 nm using a 1.0 cm quartz cell with an ultraviolet spectrophotometer (UV-2600, Shimadzu Co., Ltd., Tokyo, Japan) at sampling points every 1 nm. The fluorescence spectra were determined by Fluormax-4 (HORIBA, Ltd., Irvine, CA, USA) in a 3 cm quartz cuvette. Excitation was performed at 280 nm with a slit width of 3 nm, and emission was performed from 300 to 450 nm with a slit width of 3 nm. The CD spectra were recorded from 200 to 300 nm at a steady flow of nitrogen gas with a bandwidth set to 1 nm using a Chirascan spectropolarimeter (Chirascan, Applied Photophysics Ltd., London, UK) at room temperature and a quartz cell with 1 mm in diameter. Each UV-Vis and CD test was performed in triplicate.

## Figures and Tables

**Table 1:** The physicochemical properties of BSA ^1^.

Molecular Weight	Isoelectric Point	Grand Average of Hydropathicity	Amino Acid Residue
69,222	5.82	−0.433	583

^1^ The data are from Research Collaboratory for Structural Bioinformatics PDB.

**Table 2:** The *D_H_* of GLLCNs and GLLCNs@HDSB (*n* = 3).

Sample Name	*D_H_* (nm)	PDI	Zeta Potential (mV)
GLLCNs	135.30 ± 3.84	0.2047 ± 0.0121	−16.00 ± 0.26
GLLCNs@HDSB	207.10 ± 2.14	0.2000 ± 0.0080	−1.65 ± 0.05

**Table 3:** The results of the investigation upon GLLCNs and GLLCNs@HDSB.

Spectrum	At Low Concentrations	At High Concentrations	Conclusion
UV-Vis	No significant difference	The increase in the GLLCNs@HDSB group was greater than that in the GLLCNs group	At low concentrations GLLCNs@HDSB couldn’t inhibit the interaction between nanogels and proteins, only at high concentrations GLLCNs@HDSB produces inhibitory interactions
Fluorescence	The fluorescence quenching degree of the GLLCNs@HDSB group was weaker than that of the GLLCNs group	The fluorescence quenching intensity of the GLLCNs@HDSB group was greater than that of the GLLCNs group	
CD	The conformational changes of the GLLCNs and GLLCNs@HDSB group were not significantly different	The ellipticity values in the GLLCNs@HDSB group were smaller than those in the GLLCNs group	

## Data Availability

Not applicable.

## References

[1] Wang X., Zhang W. (2022). The Janus of Protein Corona on nanoparticles for tumor targeting, immunotherapy and diagnosis. J. Control. Release.

[2] Li Z., Xiao C., Yong T., Li Z., Gan L., Yang X. (2020). Influence of nanomedicine mechanical properties on tumor targeting delivery. Chem. Soc. Rev..

[3] De Maar J., Sofias A.M., Siegel T.P., Vreeken R.J., Moonen C., Bos C., Deckers R. (2020). Spatial heterogeneity of nanomedicine investigated by multiscale imaging of the drug, the nanoparticle and the tumour environment. Theranostics.

[4] Adityan S., Tran M., Bhavsar C., Wu S.Y. (2020). Nano-therapeutics for modulating the tumour microenvironment: Design, development, and clinical translation. J. Control. Release.

[5] Zhou Y., Dai Z. (2018). New Strategies in the Design of Nanomedicines to Oppose Uptake by the Mononuclear Phagocyte System and Enhance Cancer Therapeutic Efficacy. Chem. Asian J..

[6] Vu T.N., Le P.H.P., Pham D.N.P., Hoang T.H., Nadda A.K., Le T.S., Pham T.D. (2022). Highly adsorptive protein inorganic nanohybrid of Moringa seeds protein and rice husk nanosilica for effective adsorption of pharmaceutical contaminants. Chemosphere.

[7] Isaia H.A., Pinilla C.M.B., Brandelli A. (2020). Evidence that protein corona reduces the release of antimicrobial peptides from polymeric nanocapsules in milk. Food Res. Int..

[8] Tenzer S., Docter D., Kuharev J., Musyanovych A., Fetz V., Hecht R., Schlenk F., Fischer D., Kiouptsi K., Reinhardt C. (2013). Rapid formation of plasma protein corona critically affects nanoparticle pathophysiology. Nat. Nanotechnol..

[9] Wu G., Jiang C., Zhang T. (2018). FcγRIIB receptor-mediated apoptosis in macrophages through interplay of cadmium sulfide nanomaterials and protein corona. Ecotoxicol. Environ. Saf..

[10] Huang Y., Yamaguchi A., Pham T.D., Kobayashi M. (2017). Charging and aggregation behavior of silica particles in the presence of lysozymes. Colloid Polym. Sci..

[11] Walkey C.D., Olsen J.B., Guo H., Emili A., Chan W.C.W. (2011). Nanoparticle Size and Surface Chemistry Determine Serum Protein Adsorption and Macrophage Uptake. J. Am. Chem. Soc..

[12] Gref R., Lück M., Quellec P., Marchand M., Dellacherie E., Harnisch S., Blunk T., Müller R. (2000). ‘Stealth’ corona-core nanoparticles surface modified by polyethylene glycol (PEG): Influences of the corona (PEG chain length and surface density) and of the core composition on phagocytic uptake and plasma protein adsorption. Colloids Surf. B Biointerfaces.

[13] Kim H.R., Andrieux K., Delomenie C., Chacun H., Appel M., Desmaële D., Taran F., Georgin D., Couvreur P., Taverna M. (2007). Analysis of plasma protein adsorption onto PEGylated nanoparticles by complementary methods: 2-DE, CE and Protein Lab-on-chip^®^ system. Electrophoresis.

[14] Debayle M., Balloul E., Dembele F., Xu X., Hanafi M., Ribot F., Monzel C., Coppey M., Fragola A., Dahan M. (2019). Zwitterionic polymer ligands: An ideal surface coating to totally suppress protein-nanoparticle corona formation?. Biomaterials.

[15] Bhattacharya A., Parish C.M., Henry J., Katoh Y. (2019). High throughput crystal structure and composition mapping of crystalline nanoprecipitates in alloys by transmission Kikuchi diffraction and analytical electron microscopy. Ultramicroscopy.

[16] Spicer P.T. (2005). Progress in liquid crystalline dispersions: Cubosomes. Curr. Opin. Colloid Interface Sci..

[17] Murgia S., Biffi S., Mezzenga R. (2020). Recent advances of non-lamellar lyotropic liquid crystalline nanoparticles in nanomedicine. Curr. Opin. Colloid Interface Sci..

[18] Waheed A., Aqil M. (2021). Lyotropic liquid crystalline nanoparticles: Scaffolds for delivery of myriad therapeutics and diagnostics. J. Mol. Liq..

[19] Zhai J., Fong C., Tran N., Drummond C.J. (2019). Non-Lamellar Lyotropic Liquid Crystalline Lipid Nanoparticles for the Next Generation of Nanomedicine. ACS Nano.

[20] Gelamo E.L., Silva C.H.T.P., Imasato H., Tabak M. (2002). Interaction of bovine (BSA) and human (HSA) serum albumins with ionic surfactants: Spectroscopy and modelling. Biochim. Biophys. Acta.

[21] Gandhi S., Roy I. (2019). Synthesis and characterization of manganese ferrite nanoparticles, and its interaction with bovine serum albumin: A spectroscopic and molecular docking approach. J. Mol. Liq..

[22] Chruszcz M., Mikolajczak K., Mank N., Majorek K.A., Porebski P.J., Minor W. (2013). Serum albumins—Unusual allergens. Biochim. Biophys. Acta Gen. Subj..

[23] Fu F., Huang Z., Wang W., Ma X., Wang L., Huang Y., Hu P., Pan X., Wu C. (2021). Interaction between bovine serum albumin and Solutol^®^ HS 15 micelles: A two-stage and concentration-dependent process. J. Drug Deliv. Sci. Technol..

[24] Bondžić A.M., Jovanović D., Arsenijević N., Laban B., Pašti T.L., Klekotka U., Bondžić B.P. (2022). “Soft Protein Corona” as the Stabilizer of the Methionine-Coated Silver Nanoparticles in the Physiological Environment: Insights into the Mechanism of the Interaction. Int. J. Mol. Sci..

[25] Wang W., Zhong Z., Huang Z., Fu F., Wu L., Huang Y., Wu C., Pan X. (2022). Two Different Protein Corona Formation Modes on Soluplus^®^ Nanomicelles. Colloids Surf. B Biointerfaces.

[26] Woźniak-Budych M.J., Przysiecka Ł., Maciejewska B.M., Wieczorek D., Staszak K., Jarek M., Jesionowski T., Jurga S. (2017). Facile Synthesis of Sulfobetaine-Stabilized Cu_2_O Nanoparticles and Their Biomedical Potential. ACS Biomater. Sci. Eng..

[27] Roufik S., Gauthier S.F., Dufour A., Turgeon S.L. (2006). Interactions between Bovine β-Lactoglobulin A and Various Bioactive Peptides As Studied by Front-Face Fluorescence Spectroscopy. J. Agric. Food Chem..

[28] Ashraf S., Park J., Bichelberger M.A., Kantner K., Hartmann R., Maffre P., Said A.H., Feliu N., Lee J., Lee D. (2016). Zwitterionic surface coating of quantum dots reduces protein adsorption and cellular uptake. Nanoscale.

[29] Ojha H., Mishra K., Hassan M.I., Chaudhury N.K. (2012). Spectroscopic and isothermal titration calorimetry studies of binding interaction of ferulic acid with bovine serum albumin. Thermochim. Acta.

[30] Estephan Z.G., Schlenoff P.S., Schlenoff J.B. (2011). Zwitteration As an Alternative to PEGylation. Langmuir.

[31] Alallam B., Doolaanea A.A., Oo M.K., Nasir M.H.M., Taher M. (2021). Influence of nanoparticles surface coating on physicochemical properties for CRISPR gene delivery. J. Drug Deliv. Sci. Technol..

[32] Nikoo A.M., Kadkhodaee R., Ghorani B., Razzaq H., Tucker N. (2018). Electrospray-assisted encapsulation of caffeine in alginate microhydrogels. Int. J. Biol. Macromol..

[33] Moreira L.M., Santiago P.S., de Almeida V., Tabak M. (2008). Interaction of giant extracellular Glossoscolex paulistus hemoglobin (HbGp) with zwitterionic surfactant N-hexadecyl-N,N-dimethyl-3-ammonio-1-propanesulfonate (HPS): Effects of oligomeric dissociation. Colloids Surf. B Biointerfaces.

[34] He X., Li Q., Liu X., Wu G., Zhai G. (2015). Curcumin-Loaded Lipid Cubic Liquid Crystalline Nanoparticles: Preparation, Optimization, Physicochemical Properties and Oral Absorption. J. Nanosci. Nanotechnol..

[35] Maiorova L.A., Erokhina S.I., Pisani M., Barucca G., Marcaccio M., Koifman O.I., Salnikov D.S., Gromova O.A., Astolfi P., Ricci V. (2019). Encapsulation of vitamin B12 into nanoengineered capsules and soft matter nanosystems for targeted delivery. Colloids Surf. B Biointerfaces.

